# Elevated, FcεRI‐dependent MRGPRX2 expression on basophils in chronic urticaria

**DOI:** 10.1002/ski2.195

**Published:** 2022-12-14

**Authors:** Ewa A. Bartko, Jesper Elberling, Lars H. Blom, Lars K. Poulsen, Bettina M. Jensen

**Affiliations:** ^1^ Allergy Clinic Department of Dermatology and Allergy Copenhagen University Hospital at Gentofte Hellerup Denmark

## Abstract

**Background:**

Chronic urticaria (CU) is a skin condition driven by mast cells and basophils. The exact responsiveness profile of these cells, especially regarding the anti‐IgE treatment, Omalizumab, is not fully investigated. We sought to characterize the surface activation profile of basophils in CU during Omalizumab treatment and their responsiveness to IgE and non‐IgE stimulation.

**Methods:**

Whole blood basophils from 11 CU patients and 10 healthy controls were stimulated with either medium, anti‐IgE, fMLP, C5a, or Substance P for 30 min and characterized by flow cytometry.

**Results:**

CU patients showed a broad range of basophil count as opposed to healthy subjects. An increased number of unstimulated CD69^+^ (*p* = 0.05), but not CD63^+^ basophils was observed in CU groups in comparison to healthy. The expression of CD203c and CD200R were comparable between all groups, whilst the FcεRI was reduced with the treatment. Both IgE and non‐IgE mediated stimulations upregulated CD63, CD203c and CD200R, but not CD69 in all groups, however, no difference between the groups was observed. Among unstimulated basophils, expression of MRGPRX2 was higher in CU patients after Omalizumab treatment than in the healthy group (2.4% vs. 1.5%, *p* = 0.01). The anti‐IgE stimulation increased the number of MRGPRX2‐expressing basophils in the CU group before and after omalizumab as compared to the healthy (*p* = 0.003; *p* = 0.005). The fMLP and C5a stimulations showed a similar effect to the IgE‐mediated stimulation. The MRGPRX2 ligand, Substance P did not activate basophils.

**Conclusion:**

CU basophils show increased expression of MRGPRX2 after IgE and non‐IgE stimulation.

## Graphical abstract

The role of basophils as the effector cells in CU is widely recognized, however, several aspects of their biology, phenotype, or function, require further investigation. Basophils in CU showed resting expression of substance P receptor, MRGPRX2, that was significantly increased with IgE and non‐IgE dependent stimulations, indicating a role of the MRGPRX2 pathway in CU. Moreover, a partially preactivated profile defined by the increased expression of the CD69 activation marker was observed on unstimulated (resting) basophils in CU patients.

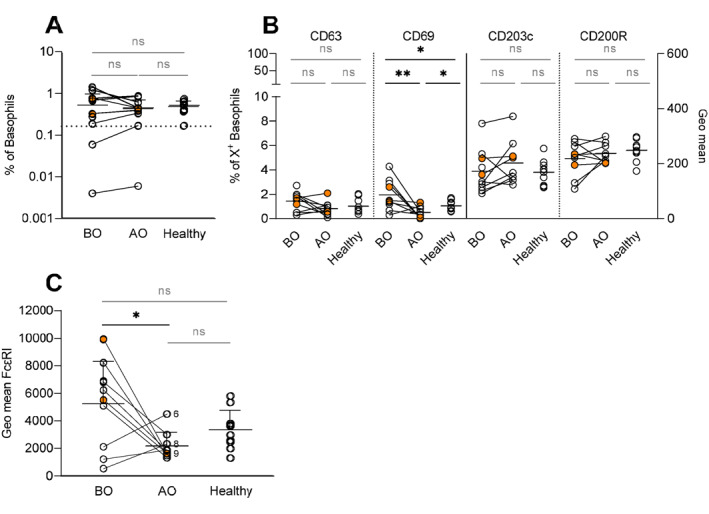

**What is already known about this topic?**

- Basophils are one of the effector cells in chronic urticaria (CU).
- Omalizumab changes basophils responsiveness in CU.

**What does this study add?**

- Basophils in CU patients showed a partially preactivated profile that Omalizumab treatment reduces.
- Basophils in CU were characterized by lower sensitivity towards IgE‐mediated stimulation.
- Increased expression of MRGPRX2 was found on resting and IgE and non‐IgE stimulated basophils from CU patients.

## 1 INTRODUCTION

Chronic urticaria (CU) is a group of skin diseases characterized by the appearance of transient pruritic wheals remaining on the skin for 6 weeks or longer.^1^ Two types of CU can be distinguished, spontaneous (CSU) and inducible (CIndU). While the key cellular players of the disease, mast cells (MCs) and basophils, are known, their responsiveness and phenotype require further investigation, especially in the context of the current treatment strategy, which includes the anti‐IgE biological drug, Omalizumab (OMZ). Although OMZ prevents the activation of MCs and basophils by binding IgE, it is not equally effective in all cases of CSU.^2^ Thereby, patients with no response to OMZ treatment are categorized as non‐responders (NR). In contrast, within the responder group, quick (QR) and slow responders (SR) are determined based on the time between the first injection and symptom release.

The current understanding of basophils' role in CSU highlights their altered responsiveness towards IgE‐mediated stimulation, lowered peripheral cell number (basopenia), and transmigration to the skin wheals.^3^, ^4^, ^5^ While stimulation of basophils in CSU with Substance P (SP) induces marked histamine release,^1^, ^6^ the anaphylatoxin, complement component C5a results in reduced mediator release.^1^ The expression of SP receptor—NK1R has been found elevated on basophils in CSU patients, supporting the effect of SP.^6^ Additionally, another SP receptor, Mas‐related G‐protein coupled receptor member X2 (MRGPRX2), showed higher expression on MCs in lesioned skin of CSU patients,^7^ but to our knowledge, this has not been investigated in CU basophils.

Characterization of basophil activation status either by determining the released mediators or by detecting molecules that appear on the plasma membrane upon fusion of secretory vesicles allows for a better understanding of basophil phenotype in a disease. Thus, the expression of activation receptors, for example, CD63, CD203c, CD69 has been investigated in several chronic urticaria studies.^8^, ^9^, ^10^, ^11^, ^12^ The increased baseline levels of CD63 and CD69 and comparable CD203c values contrary to non‐allergic controls were defined by Vasagar et al.,^11^ whereas Christensen et al. showed augmented expression of CD203c and CD63.^10^

In the present study, we aimed to investigate the expression of CD63, CD69, CD203c, CD200R, MRGPRX2, and FcεRI on resting and IgE or non‐IgE‐activated basophils from CU patients. Additionally, we sought to determine an effect of OMZ treatment on these markers.

## 2 MATERIALS AND METHODS

For a detailed description of the methods, please refer to the Supporting information.

### 2.1 Study design

Eleven CU patients and 10 healthy subjects were enroled in the study. Whole blood samples were collected before the first OMZ administration (BO) and 12 weeks (3 dosages) after initiation of OMZ treatment (AO). Healthy participants were subjected to one blood sampling. Patients were classified as either QR with an improvement < 1 month from the first injection, as SR with improvement ≥ 1 month from the first injection, or NR without improvement ≤ 3 months from the first injection.

### 2.2 Flow cytometric analysis of basophils

Heparinized whole blood samples were used for the investigation of surface receptors. The blood was analyzed within 4 h after collection. In brief, whole blood was simultaneously activated and stained with antibodies for 30 min at 37°C, followed by erythrolysis, fixation, and a readout on the flow cytometer.

### 2.3 Statistical analysis

Statistical analysis was performed using GraphPad Prism software, 9.0 (GraphPad Software). An unpaired *t*‐test was utilized to test for significance. A two‐sided α‐level <0.05 was considered statistically significant (*) *p* < 0.05 and (**) *p* < 0.01.

## 3 RESULTS

### 3.1 Patients' characteristics

The healthy control group (*n* = 10) almost entirely matched the CU group (*n* = 11) (Table 1). Nine out of 11 CU patients were diagnosed with CSU and CSU with concomitant CIndU, while two CIndU patients were diagnosed with urticaria factitia and cholinergic urticaria (Table S1). Three patients were classified as SR, whereas the remaining seven, except for one CIndU NR, were classified as QR (Table S1).

**TABLE 1:** Demographic features of the study population

Demographic features	CU (*n* = 11)	Healthy (*n* = 10)	*p* value
Age (y)	38.5 (22–58)	36.1 (25–53)	0.6348
Sex F (M); % F	8 (3); 73	8 (2); 80	0.7132
Duration of disease (y)	4 (0.5–11)	n/a	
CSU; no. (%)	4 (36)	n/a	
CSU + CIndU no. (%)	5 (45)	n/a	
CIndU no. (%)	2 (18)	n/a	
Responder—quick/slow (QR/SR)	6/3	n/a	
Non‐responder (NR)	1	n/a	

*Note*: Age, diagnosis, and disease duration as mean and range/percentage (parenthesis) in CU (*n* = 11) and healthy (*n* = 10) individuals. Statistics were calculated by unpaired *t*‐test; CU, CSU, and CIndU define chronic, chronic spontaneous, and chronic inducible urticaria, respectively, n/a, not applicable; Patient no. 10 did not receive OMZ treatment, thus, he/she was excluded from the QR/SR/NR classification. CIndU patients are marked in orange.

### 3.2 Basophil cell count

**FIGURE 1:**
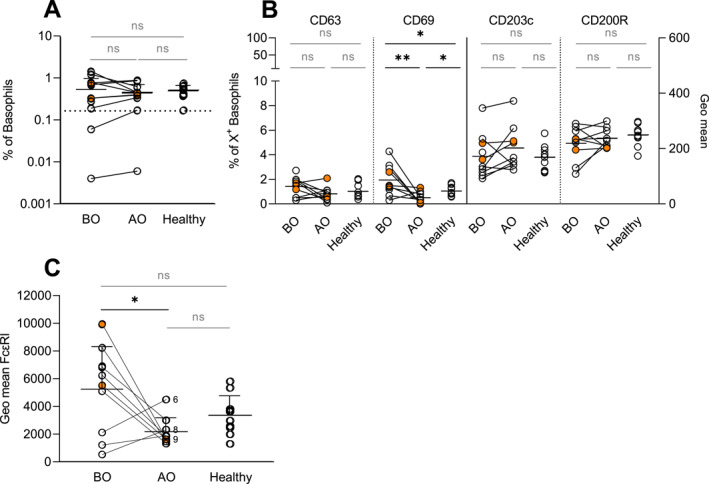
Resting expression of activation, inhibitory, and FcαRI on basophils. Basophils at the resting state were investigated for surface expression of CD63, CD69, CD203c, CD200R, and FcεRI. (a) Basophil count; dotted line shows the cut‐off value of 0.164. (b) Resting expression of CD63, CD69, CD203c, and CD200R; % of CD63^+^ and CD69^+^ basophils—left axis and GeoMean of CD203c and CD200R—right axis. (c) Resting expression of FcεRI. BO and AO refer to patients' groups before and after Omalizumab treatment, respectively; CIndU patients are marked in orange; BO (*n* = 10), AO (*n* = 9) and healthy (*n* = 10). Patient no. 3 was excluded from b and c, due to being basopenic; Statistics applied—unpaired *t*‐test with a two‐sided α‐level, <0.05 considered as significant; ns, non‐significant, *p* > 0.05, *p ≤ 0.05, **p ≤ 0.01 Mean ± SD; CIndU, chronic inducible urticaria; SD, standard deviation.

Initially, we focussed on the basopenic theory of CU patients. CU patients showed a broad range of basophil count ranging from 0.004% to 1.44% of single cells (Figure 1a). Two basopenic patients were identified, one in QR and one in SR/NR group.

### 3.3 Expression of activation, inhibitory and FcεRI receptors on resting, IgE, and non‐IgE stimulated basophils

We next investigated if OMZ treatment modulated the basophil activation profile. The resting expression profile of basophils (CD123^+^CRTH2^+^, Figure S1) showed no expression (<2%) of CD63 in either of the groups and higher expression of CD69 in the BO group compared to AO (*p* = 0.005) and healthy (*p* = 0.05) (Figure 1b). Moreover, fewer basophils expressing CD69 were observed in the AO than in healthy individuals (*p* = 0.02) (Figure 1b). Interestingly, comparable levels of the well‐recognized basophil activation markers CD203c and CD200R were measured in all groups (Figure 1b). As expected, FcεRI expression on resting basophils was reduced by the OMZ treatment (*p* = 0.01). Furthermore, a trend of elevated expression of FcεRI was found in the BO group as opposed to healthy individuals (*p* = 0.09) (Figure 1c).

**FIGURE 2:**
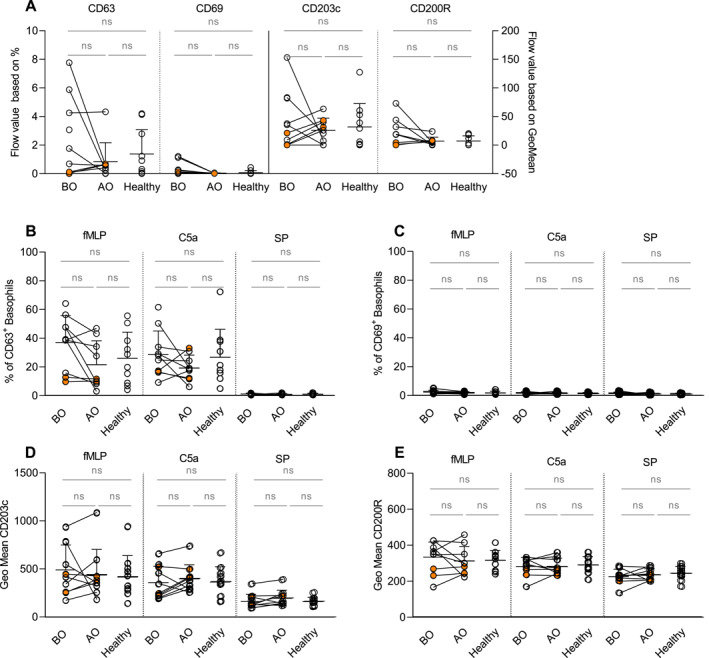
IgE and non‐IgE mediated activation of basophils—an expression of activation and inhibitory receptors. Basophils were activated with serial dilution of anti‐IgE (4–4000 ng/ml), fMLP, C5a, and SP. (a) Flow values of percentage (CD63^+^ and CD69^+^) and GeoMean (CD203c and CD200R) of basophils. (b–e) Non‐IgE induced expression of CD63 (b) and CD69 (c) and GeoMean of CD203c (d) and CD200R (e). BO and AO refer to patients' groups before and after Omalizumab treatment, respectively; CIndU patients are marked in orange; BO (*n* = 10), AO (*n* = 9), and healthy (*n* = 10); Statistics applied—unpaired *t*‐test; ns, non‐significant; Mean ± SD; CIndU, chronic inducible urticaria; SD, standard deviation.

IgE–mediated stimulation with serial dilution of anti‐IgE resulted in a dose‐dependent increase in the percentage of CD63^+^ basophils and elevated expression of CD203c and CD200R (Figure S2, A–D). However, no changes in the expression of receptors were observed between the groups. Interestingly, OMZ altered the expression pattern for all receptors from scattered in the BO group to stacked, indicating an effect of the treatment on receptor expression (Figure 2a).

fMLP and C5a stimulations increased the percentage of CD63^+^ basophils and induced upregulation of CD203c and CD200R in all groups (Figure S3, A,C,D). OMZ tended to reduce the number of CD63 expressing basophils induced by fMLP (*p* = 0.075) and by C5a (*p* = 0.14) (Figure 2b). The upregulated expressions of CD203c and CD200R were comparable for both stimulants between all groups, and there were no changes in the expression of CD69 (Figure 2c–e). SP failed to trigger basophil activation (Figure 2).

### 3.4 Expression of MRGPRX2 on resting, IgE, and non‐IgE activated basophils

**FIGURE 3:**
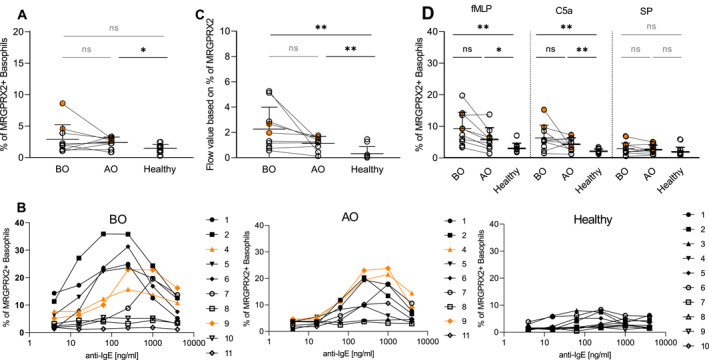
Resting, IgE and non‐IgE mediated expression of MRGPRX2. Resting, anti‐IgE (4–4000 ng/ml), fMLP, C5a, and SP stimulated basophils were investigated for surface expression of MRGPRX2. (a) Resting expression of MRGPRX2—% of MRGPRX2^+^ basophils. (b) Dose‐dependent effect of IgE—mediated stimulation on the % of MRGPRX2^+^ basophils—*X*‐axis depicts anti‐IgE (4–4000 ng/ml), and *Y*‐axis shows the change in the %. (c) Flow values for the % of MRGPRX2^+^ basophils. (d) Non‐IgE mediated % of MRGPRX2^+^ basophils. BO and AO refer to patients' groups before and after Omalizumab treatment, respectively; CIndU patients are marked in orange; BO (*n* = 10), AO (*n* = 9) and healthy (*n* = 10); Statistics applied—unpaired *t*‐test with a two‐sided α‐level, <0.05 considered as significant; ns, non‐significant, *p* > 0.05, *p ≤ 0.05;**p ≤ 0.01 Mean ± SD; CIndU, chronic inducible urticaria; SD, standard deviation.

We then evaluated the expression of MRGPRX2 on resting basophils. Contrary to healthy, we found a higher percentage of MRGPRX2^+^ basophils in the OMZ‐treated group (*p* = 0.01) (Figure 3a). Interestingly, OMZ altered the resting expression of MRGPRX2 on basophils in CU patients (*p* = 0.08) similarly to the flow values of the activation and inhibitory receptors. As we expected, no expression (*x̄* = 1.5%) of MRGPRX2 was found on resting healthy basophils (Figure 3a).

IgE–mediated stimulation induced a marked, dose‐dependent increase in the percentage of MRGPRX2^+^ basophils in the BO group (Figure 3b). The expression of MRGPRX2 on basophils was higher in the BO and AO groups compared to healthy (*p* = 0.003; *p* = 0.005). Moreover, OMZ tended to reduce the percentage of MRGPPRX2^+^ basophils (*p* = 0.08) (Figure 3c).

The fMLP stimulation triggered the highest increase in the percentage of MRGPRX2^+^ basophils in all groups, followed by C5a and SP, with the latter triggering no changes. The most pronounced difference was observed for the BO group in comparison to the healthy (Figure S3,E). The fMLP stimulation resulted in a higher frequency of MRGPRX2^+^ basophils in BO (*p* = 0.002) and AO (*p* = 0.04) groups compared to healthy (Figure 3d). The same pattern was observed for the C5a stimulation (BO—*p* = 0.005 and AO—*p* = 0.004 vs. healthy). OMZ treatment tended to normalize this; however not reaching statistical significance for any stimulant. The SP activation was ineffective in inducing MRGPRX2 expression in all groups (Figure 3d).

## 4 DISCUSSION

In this study, we sought to evaluate the effect of OMZ treatment on basophils' responsiveness and activation status. We confirmed the constitutive expression of CD203c and CD200R and no expression of CD63 and CD69 on resting basophils of healthy individuals. However, contrary to Christensen et al.^10^ and Chen et al.,^9^ we did not observe the expression of CD63 in resting basophils of CU patients, whereas the CD203c expression showed comparable levels to healthy. Differences between the studies could stem from staining procedures, where basophils were stained with either fluorophore‐labelled anti‐FcεRI or anti‐IgE that could potentially activate cells. Additionally, in the study by Chen et al., enrichment of basophils was utilized, known to preactivate basophils.^9^ The increased number of basophils expressing CD69 in CU suggests an in vivo stimulation which could be induced either by dysregulated intracellular pathways governing an expression of the receptor or by increased concentrations of molecules priming basophil responsiveness such as IL‐3. The lower expression of CD69 on basophils in CU induced by OMZ might indicate a link between the activation marker and the FcERI‐IgE pathway, as OMZ reduced levels of both in CU patients. Additionally, it implies that OMZ decreases the activation profile of basophils. Although the CD200R was not reduced, a tendency of decreased expression was observed in a few patients indicating ameliorated inhibitory properties of basophils in these CU patients, which could also be a reason for the elevated expression of CD69 on resting basophils.

The increased CD63 expression induced by lower anti‐IgE concentrations and the higher CD63 flow values suggests a more responsive profile of IgE‐mediated activation of basophils in CU. This could be explained by a disrupted intracellular signalling pathway with increased phosphorylation of SYK needed for the propagation of the signal and/or reduced function of inhibitory molecules such as SHIP1/2.^13^, ^14^ As the CD63 upregulation pathway differs from CD203c,^15^, ^16^ the CD203c—based sensitivity profile was also investigated. No difference between basophils in CU and healthy in regards to CD203c expression was found, implying that two different modes of basophil activation are independently affected in CU. Additionally, a tendency of higher CD200R expression in CU basophils implies that not only are inhibitory mechanisms still effective, but they also are more sensitive in contrary to healthy basophils. The GPCR—mediated activation with fMLP and C5a upregulated CD63, CD203c, and CD200R in all groups. As fMLP stimulation requires a more robust cytosolic response than IgE‐mediated stimulation,^17^ the amplified response of CU basophils via this pathway might suggest either higher reserves of Ca^2+^, their faster disposition, or both. In contrast to Luquin et al.,^1^ we did not observe reduced responsiveness of basophils in urticaria patients to C5a stimulation, possibly due to a 10 times higher concentration of C5a in this study. Correspondingly to Deza et al.,^2^ we measured higher FcεRI expression on resting basophils in CU, with QR presenting increased values contrary to SR. Even though NR basophils showed FcεRI levels similar to the values of QR, they did not respond to the OMZ treatment, suggesting an alternative mechanism driving the disease.

To our knowledge, we demonstrated for the first time a trend of elevated expression of MRGPRX2 on resting CU basophils compared to the healthy. This, however, did not correspond with the SP activation of basophils, which failed to induce degranulation and was in contrast to the study by Zheng et al.^6^ We hypothesized that even though the number of MRGPRX2^+^ basophils was higher in CU, the expression of the receptor must have been insufficient to result in cell degranulation. Additionally, this might indirectly suggest an absence of an alternative pathway for SP activation, for example, NK1R. IgE and non‐IgE mediated stimulations further expanded the resting expression of MRGPRX2 on basophils in CU. The anti‐IgE activation resulted in the MRGPRX2 upregulation, which profile followed the pattern of CD63 upregulation. However, contrary to CD63, the pattern of the dose‐dependent curves of MRGPRX2 differed drastically in the healthy subjects. This marked difference between MRGPRX2 and CD63 highlights the role of the former in activating basophils in CU. Moreover, intracellular components responsible for the upregulation of MRGPRX2 might be dysregulated in CU basophils since in healthy subjects, the upregulation of CD63 and MRGPRX2, though showing comparable sensitivity towards a stimulant, resulted in the marked upregulation of the former, however marginal of the latter. Another explanation for the increased number of MRGPRX2^+^ basophils in CU could be the matrix surrounding basophils, that is, serum, which components might prime cells to respond to the IgE‐mediated stimulation in a certain way. Thus, the mechanism linking the IgE‐FcERI pathway with MRGPRX2 in CU depicts basophils with a decreased threshold for the activation via the IgE pathway leading to higher upregulation of MRGPRX2. In contrast to Wedi et al.,^18^ we demonstrated no (*x̄* = 1.5%) expression of MRGPRX2 on healthy basophils. This, however, partially supports results from Sabato et al.,^19^, ^20^ showing only a minority of basophils in healthy subjects expressing MRGPRX2. Moreover, our results align with the study by Fujisawa et al.,^7^ in which elevated MRGPRX2 expression was found on MCs in lesioned skin of CU patients. In addition, an increased serum level of soluble MRGPRX2 correlated with CSU severity showing strong evidence as a marker for disease severity.^21^ These findings and our data suggest an involvement of the pseudo‐allergen pathway in the regulation of effector cell activation in CU patients and the disease profile.

The major limitation of this study was the low number of participants and the heterogeneity of patients. The latter justifies the broad range of basophil count in patients, which is known to depend on disease severity. Thus, this study should be considered a foundation for future projects focussing on MRGPRX2 signalling and the sensitivity profile of basophils in urticaria patients.

In summary, CU patients presented a broad spectrum of basophil count and a higher anti‐IgE‐induced sensitivity profile of basophils. Furthermore, the resting expression of MRGPRX2 on CU basophils was significantly upregulated upon IgE and non‐IgE mediated stimulations.

## CONFLICT OF INTEREST

All authors declare no conflicts of interest.

## AUTHOR CONTRIBUTIONS

**Ewa A. Bartko**: Conceptualization (Equal); Data curation (Lead); Formal analysis (Lead); Investigation (Lead); Methodology (Equal); Project administration (Lead); Visualization (Lead); Writing – original draft (Lead); Writing – review & editing (Lead). **Jesper Elberling**: Conceptualization (Equal); Data curation (Supporting); Formal analysis (Supporting); Methodology (Supporting); Supervision (Equal); Visualization (Supporting); Writing – original draft (Supporting); Writing – review & editing (Supporting). **Lars H. Blom**: Data curation (Supporting); Formal analysis (Supporting); Methodology (Supporting); Visualization (Supporting); Writing – original draft (Supporting); Writing – review & editing (Supporting). **Lars K. Poulsen**: Conceptualization (Equal); Data curation (Supporting); Formal analysis (Supporting); Funding acquisition (Lead); Methodology (Supporting); Project administration (Supporting); Supervision (Equal); Visualization (Supporting); Writing – original draft (Supporting); Writing – review & editing (Supporting). **Bettina M. Jensen**: Conceptualization (Equal); Data curation (Supporting); Formal analysis (Supporting); Funding acquisition (Supporting); Methodology (Supporting); Project administration (Supporting); Supervision (Equal); Visualization (Supporting); Writing – original draft (Supporting); Writing – review & editing (Supporting).

## ETHICS STATEMENT

The study was approved by the Ethical Committee of the Capital Region of Denmark (H‐20034184) and by the Danish Data Protection Agency.

## Supporting information

Supporting Information S1

## Data Availability

The data that support the findings of this study are available on request from the corresponding author. The data are not publicly available due to privacy or ethical restrictions.

## References

[1] Luquin E , Kaplan AP , Ferrer M . Increased responsiveness of basophils of patients with chronic urticaria to sera but hypo‐responsiveness to other stimuli. Clin Exp Allergy. 2005;35(4):456–60. 10.1111/j.1365-2222.2005.02212.x 15836753

[2] Deza G , Bertolin‐Colilla M , Pujol R , Curto‐Barredo L , Soto D , Garcia M , et al. Basophil Fc3RI expression in chronic spontaneous urticaria: a potential immunological predictor of response to omalizumab therapy. Acta Derm Venereol. 2017;97(6):698–704. 10.2340/00015555-2654 28303277

[3] Saini S . Chronic spontaneous urticaria: etiology and pathogenesis. Immunol Allergy Clin. 2014;34(1):33–52. 10.1016/j.iac.2013.09.012 PMC11218737 24262688

[4] Rorsman H . Basophilic leucopenia in different forms of urticaria. Allergy. 1962;17(2):168–84. 10.1111/j.1398-9995.1962.tb02937.x 14493754

[5] Elias J , Boss E , Kaplan AP . Studies of the cellular infiltrate of chronic idiopathic urticaria: prominence of T‐lymphocytes, monocytes, and mast cells. J Allergy Clin Immunol. 1986;78(5):914–8. 10.1016/0091-6749(86)90240-X 3491100

[6] Zheng W , Wang J , Zhu W , Xu C , He S . Upregulated expression of substance P in basophils of the patients with chronic spontaneous urticaria: induction of histamine release and basophil accumulation by substance P. Cell Biol Toxicol. 2016;32(3):217–28. 10.1007/s10565-016-9330-4 27147256

[7] Fujisawa D , Kashiwakura J , Kita H , Kikukawa Y , Fujitani Y , Sasaki‐Sakamoto T , et al. Expression of Mas‐related gene X2 on mast cells is upregulated in the skin of patients with severe chronic urticaria. J Allergy Clin Immunol. 2014;134(3):622–33.e9. 10.1016/j.jaci.2014.05.004 24954276

[8] Rauber MM , Pickert J , Holiangu L , Möbs C , Pfützner W . Functional and phenotypic analysis of basophils allows determining distinct subtypes in patients with chronic urticaria. Allergy Eur J Allergy Clin Immunol. 2017;72(12):1904–11. 10.1111/all.13215 28585360

[9] Chen Q , Zhai Z , Xu J , Chen W , Chen S , Zhong H , et al. Basophil CD63 expression in chronic spontaneous urticaria: correlation with allergic sensitization, serum autoreactivity and basophil reactivity. J Eur Acad Dermatol Venereol. 2017;31(3):463–8. 10.1111/jdv.13912 27518369

[10] Christensen CU , Vestergaard C , Hoffmann HJ . Activation markers CD63 and CD203c are upregulated in chronic urticaria. Ann Dermatol. 2013;25(4):522–3. 10.5021/ad.2013.25.4.522 24371413 PMC3870234

[11] Vasagar K , Vonakis BM , Gober LM , Viksman A , Gibbons SP , Saini SS . Evidence of in vivo basophil activation in chronic idiopathic urticaria. Clin Exp Allergy. 2006;36(6):770–6. 10.1111/j.1365-2222.2006.02494.x 16776678

[12] Yasnowsky KM , Dreskin S , Efaw B , Schoen D , Vedanthan P , Alam R , et al. Chronic urticaria sera increase basophil CD203c expression. J Allergy Clin Immunol. 2006;117(6):1430–4. 10.1016/j.jaci.2006.02.016 16751009

[13] Vonakis BM , Vasagar K , Gibbons SP , Gober L , Sterba PM , Chang H , et al. Basophil FcεRI histamine release parallels expression of Src‐homology 2‐containing inositol phosphatases in chronic idiopathic urticaria. J Allergy Clin Immunol. 2007;119(2):441–8. 10.1016/j.jaci.2006.09.035 17125820

[14] Saini SS , Paterniti M , Vasagar K , Gibbons SP , Sterba PM , Vonakis BM . Cultured peripheral blood mast cells from chronic idiopathic urticaria patients spontaneously degranulate upon IgE sensitization: relationship to expression of Syk and SHIP‐2. Clin Immunol. 2009;132(3):342–8. 10.1016/j.clim.2009.05.003 19477690 PMC2720433

[15] Hennersdorf F , Florian S , Jakob A , Baumgartner K , Sonneck K , Nordheim A , et al. Identification of CD13, CD107a, and CD164 as novel basophil‐activation markers and dissection of two response patterns in time kinetics of IgE‐dependent upregulation. Cell Res. 2005;15(5):325–35. 10.1038/sj.cr.7290301 15916720

[16] MacGlashan D . Expression of CD203c and CD63 in human basophils: relationship to differential regulation of piecemeal and anaphylactic degranulation processes. Clin Exp Allergy. 2010;40(9):1365–77. 10.1111/j.1365-2222.2010.03572.x 20633031 PMC2927965

[17] Warner JA , MacGlashan DW . Signal transduction events in human basophils. A comparative study of the role of protein kinase C in basophils activated by anti‐IgE antibody and formyl‐methionyl‐leucyl‐phenylalanine. J Immunol. 1990;145:1897–905. 1697313

[18] Wedi B , Gehring M , Kapp A . The pseudoallergen receptor MRGPRX2 on peripheral blood basophils and eosinophils: expression and function. Allergy Eur J Allergy Clin Immunol. 2020;75(9):2229–42. 10.1111/all.14213 32003863

[19] Sabato V , Elst J , Van Houdt M , Bridts C , Mertens C , Ebo DG . Surface expression of MRGPRX2 on resting basophils: an area of controversy. Allergy Eur J Allergy Clin Immunol. 2020;75(9):2421–2. 10.1111/all.14252 32929729

[20] Sabato V , Van Gasse A , Cop N , Claesen K , Decuyper II , Faber MA , et al. The Mas‐related G protein‐coupled receptor MRGPRX2 is expressed on human basophils and up‐regulated upon activation. J Allergy Clin Immunol. 2017;139(2):AB168. 10.1016/j.jaci.2016.12.550

[21] Cao TBT , Cha HY , Yang EM , Ye YM . Elevated MRGPRX2 levels related to disease severity in patients with chronic spontaneous urticaria. Allergy Asthma Immunol Res. 2021;13(3):498–506. 10.4168/aair.2021.13.3.498 33733642 PMC7984951

