# Supplementary material for: Elevated, FcεRI‐dependent MRGPRX2 expression on basophils in chronic urticaria
Source: Skin Health Dis. 2022 Dec 14;3(3):e195. doi: 10.1002/ski2.195 (PMC10233071; doi:10.1002/ski2.195)
Supplement: Supplementary file 1 — Supporting Information S1 [file SKI2-3-e195-s001.docx]

**Supplementary**

**Materials and Methods**

Study design

CU patients were recruited from the Urticaria Clinic at the Department of Dermatology at Gentofte Hospital, Denmark, from March to December 2021. The Ethical Committee of the Capital Region of Denmark (H-20034184) and the Danish Data Protection Agency approved the study. The written, signed consent forms were obtained from all study subjects.

A physician diagnosed patients on the first visit to the Urticaria Clinic. The diagnosis of patients followed an internal protocol based on international guidelines.

One CU patient had blood collected only before Omalizumab (OMZ) administration due to a change in the treatment plan. The clinical outcome of the treatment, described as a decrease in the severity of symptoms, was defined by the velocity at which patients responded to the drug. This was verified during the AO visit, where a doctor interviewed a patient to assess the effect of the treatment. Improvement was defined as clear or almost clear of any itch, wheal, and/or flare reaction.

Flow cytometric analysis of basophils

***Surface staining*** *-* 100 µL of whole blood was simultaneously stained with BV650 anti–CD123 (7G3, BD), PerCP efluor 710 anti–CD200R (OX108, Invitrogen), BV480 anti–CD203c (NP4D6, BD), FITC anti–CD63 (H5C6, BD), PE anti–CD69 (FN50, BD), PE-CF594 anti–CRTH2 (BM16, BD), PE anti–MRGPRX2 (K125H4, Biolegend), and stimulated with either medium (RPMI1640 + 0.5% (v/v) HSA; Sigma-Aldrich, USA, CSL Behring GmbH, Germany) serial dilution of polyclonal goat anti-human IgE (ε) (anti–IgE), 4 – 4000ng/mL, (WVR, USA), 0.5 µg/mL formyl-methionyl-leucyl-phenylalanine (fMLP) (Sigma-Aldrich, US), 1000 ng/mL C5a, (R&D, USA) or 10 µM Substance P (Sigma-Aldrich, USA) for 30 min at 37°C in a water bath. Incubation was followed by 10 min fixation and erythrolysis at RT with BD FACS lysing solution (BD). Next, cells were stained for 30 min at 4°C with BV421 anti–FcεRIα (AER–37, CRA-1, Biolegend), followed by washing and analysis with LSR II Fortessa flow cytometer (BD).

Basophils were gated as CD123^+^CRTH2^+^ cells (Figure S1). Cells stimulated with anti–IgE 1000 ng/mL were used as FMO controls for CD69 and MRGPRX2. FMO gate was set at 1%. Expression of receptors was determined as either the percentage of basophils positive for a marker based on an FMO control - X^+^ basophils or geometric mean of fluorescence intensity – GeoMean. The former was applied to receptors with a low expression, such as CD63, CD69, and MRGPRX2, whereas the latter was utilized for constitutively expressed receptors, such as CD203c, CD200R, and FcεRI. The cut-off value for the anti-IgE releaser phenotype was 10% for CD63 and CD69. Data were analyzed with FlowJo software version 10 (TreeStar, Ashland, OR, USA).

Basophil responsiveness profile – flow values

To investigate the profile of IgE-dependent responsiveness, basophils were stimulated with serial dilution of anti-IgE. This resulted in the generation of curves that showed a dose-dependent response for some receptors. To address this, the flow value was calculated for each receptor with the following equation:

Flow value = C6-1×Y6 + C5-1×Y5 + C4-1×Y4 + C3-1×Y3 + C2-1×Y2 + C1-1×Y1

where C6–C1 indicates the anti-IgE concentration in ng/mL (4, 16, 63, 250, 1000, 4000 ) and Y6–Y1 is either percentage of receptor-positive basophils or GeoMean value corrected for the background, which is the GeoMean of resting basophils

Basophil cell count

The cut-off value for basopenia was calculated as follows:

$$cut-off=\mu-2\sigma$$

*where* $\mu$ *indicates the mean value of the healthy group and* $2\sigma$ *represents 2 times SD values*

Two SDs correspond to a 95% confidence interval. Values below the cut-off indicate basopenia.

**Tabel and figures**

|  | **Diagnosis** | **Sex** | **Age** | **Clinical outcome** |
| --- | --- | --- | --- | --- |
| **1** | CSU + CIndU | M | 38 | QR |
| **2** | CSU | F | 56 | QR |
| **3** | CSU | F | 49 | SR |
| **5** | CSU + CIndU | M | 26 | QR |
| **6** | CSU | F | 58 | SR |
| **7** | CSU | F | 29 | QR |
| **8** | CSU | F | 28 | SR |
| **10** | CSU | F | 26 | n/a |
| 11 | CSU + CIndU | F | 41 | QR |
| **4** | CIndU (urticaria factitia) | F | 46 | QR |
| **9** | CIndU (cholinergic urticaria) | M | 27 | NR |

**Table 1S. Clinical outcome**

The table depicts the clinical outcome of the treatment with sex, age, and diagnosis for the individual patient. Slow and non-responders are underlined. CSU and CSU + CIndU patients are groups above the black line, while the CIndU patients can be found below. CU, CSU, and CIndU define chronic, chronic spontaneous, and chronic inducible urticaria, respectively; QR – quick responder, SR – slow responder, and NR – non-responder, n/a – not applicable.

**
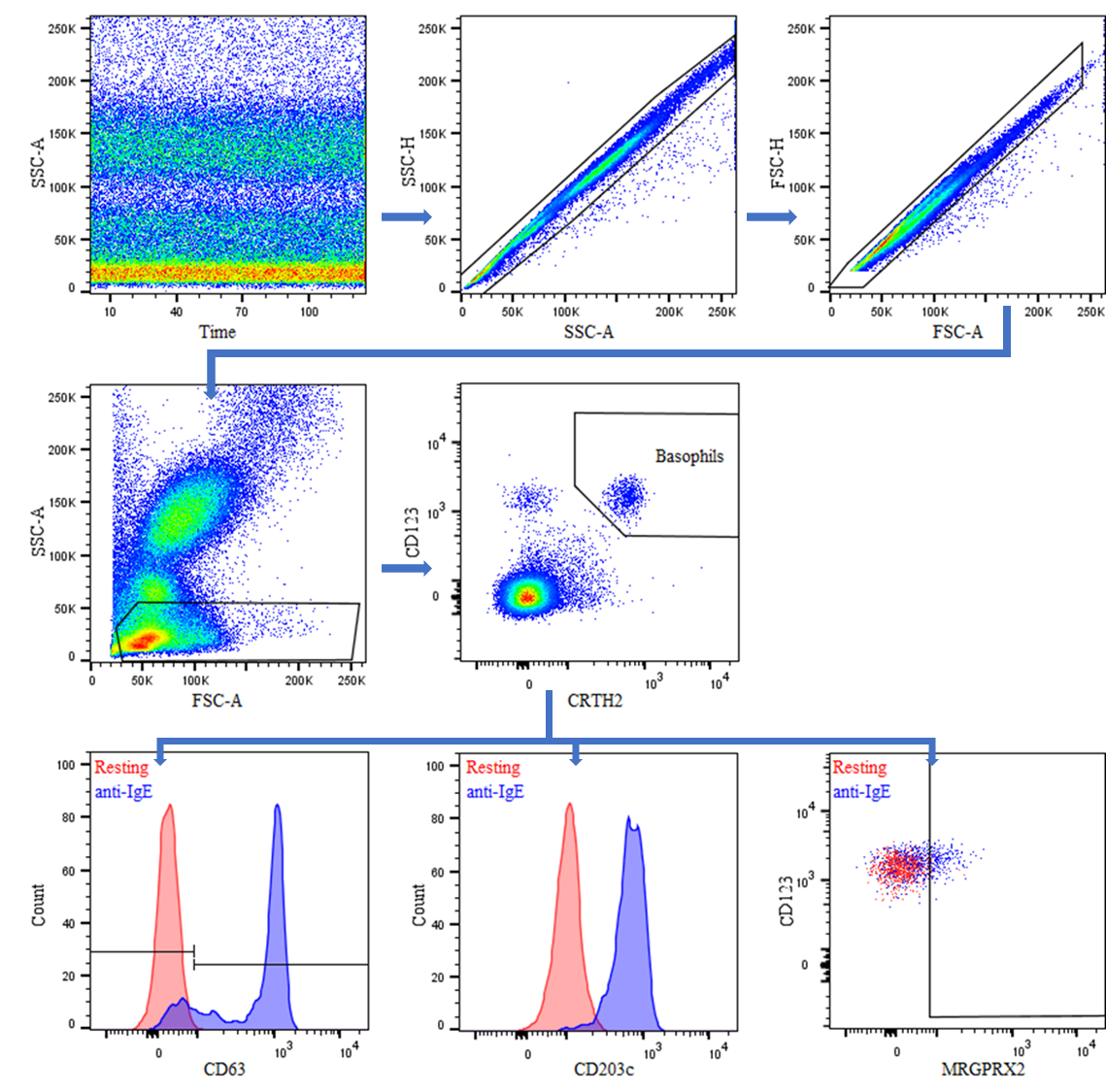
**

**Figure 1S. Gating strategy of basophils**

Whole blood basophils were gated: SSC-A vs. time, SSC-A vs. SSC-H, FSC-A vs. FSC-H, FSC-A vs. SSC-A, and CRTH2 vs. CD123 (basophils gate). Within basophils’ gate subsequent gating strategies were applied: % of CD63^+^ basophils (histogram), GeoMean of CD203c (histogram) and % of CD123^+^MRGPRX2^+^ basophils.

**Figure 2S. IgE-mediated activation of basophils – IgE-mediated dose-dependent change in receptor expression.**

Basophils were activated with serial dilution of anti-IgE (4-4000 ng/mL) – X-axis. Change in the investigated receptor is depicted on the Y-axis. **A.** % of CD63^+^ basophils, **B.** % of CD69^+^ basophils, **C.** GeoMean of CD203c, and **D.** GeoMean of CD200R.

BO and AO refer to patients’ groups before and after Omalizumab treatment, respectively; BO (n=10), AO (n=9), and healthy (n=10)

**Figure 3S. Non-IgE mediated activation of basophils**

Basophils were activated with fMLP, C5a, and SP. The surface expression of CD63, CD203c, CD69, CD200R, and MRGPRX2 was determined. **A**. % of CD63^+^ basophils **B**. % of CD69^+^ basophils **C**. GeoMean of CD203c **D**. GeoMean of CD200R **E**. % of MRGPRX2^+^ basophils

BO and AO refer to patients’ groups before and after Omalizumab treatment, respectively; BO (n=10), AO (n=9), and healthy (n=10); Statistics applied – unpaired t-test with a two-sided α-level, < 0.05 considered as significant; ns – non-significant, *p* > 0.05, * - *p* ≤ 0.05, ** - *p* ≤ 0.01, *** - *p* ≤ 0.001, **** - *p* ≤ 0.0001: Mean ± SD; SD – standard deviation
